# HUR protects *NONO* from degradation by mir320, which is induced by p53 upon UV irradiation

**DOI:** 10.18632/oncotarget.13002

**Published:** 2016-11-01

**Authors:** Luigi Alfano, Caterina Costa, Antonella Caporaso, Dario Antonini, Antonio Giordano, Francesca Pentimalli

**Affiliations:** ^1^ Oncology Research Center of Mercogliano (CROM), Istituto Nazionale Per Lo Studio E La Cura Dei Tumori “Fondazione Giovanni Pascale”, IRCCS, Naples, 80131, Italy; ^2^ Department of Medicine, Surgery and Neuroscience, University of Siena and Istituto Toscano Tumori (ITT), Siena, 53100, Italy; ^3^ IRCCS SDN, Naples, 80143, Italy; ^4^ Sbarro Institute for Cancer Research and Molecular Medicine, Center for Biotechnology, College of Science and Technology, Temple University, Philadelphia PA, 19122, USA

**Keywords:** DNA damage response, NONO, p53, HUR, mir320a

## Abstract

UV radiations challenge genomic stability and are a recognized cancer risk factor. We previously found that the RNA-binding protein NONO regulates the intra-S phase checkpoint and its silencing impaired HeLa and melanoma cell response to UV-induced DNA damage. Here we investigated the mechanisms underlying NONO regulation upon UVC treatment. We found that UVC rays induce the expression of mir320a, which can indeed target *NONO.* However, despite mir320a induction, NONO mRNA and protein expression are not affected by UVC. We found through RNA immunoprecipitation that UVC rays induce the ubiquitous RNA-binding protein HUR to bind *NONO* 5′UTR in a site overlapping mir320a binding site. Both HUR silencing and its pharmacological inhibition induced *NONO* downregulation following UVC exposure, whereas concomitant mir320a silencing restored *NONO* stability. UVC-mediated mir320a upregulation is triggered by p53 binding to its promoter, which lies within a region marked by H3K4me3 and H3K27ac signals upon UVC treatment. Silencing mir320a sensitizes cells to DNA damage. Overall our findings reveal a new mechanism whereby HUR protects *NONO* from mir320-mediated degradation upon UVC exposure and identify a new component within the complex network of players underlying the DNA damage response adding mir320a to the list of p53-regulated targets upon genotoxic stress.

## INTRODUCTION

Safeguarding genome integrity is crucial to prevent the accumulation of cancer promoting genetic alterations. By directly causing harmful DNA lesions [1], UV radiations are among the major environmental threats to genomic stability. Indeed, UV radiations represent the main risk factor for skin cancer, including melanoma.

Cells respond to UV-induced DNA damage by activating a finely tuned cascade of events which include the activation of cell-cycle checkpoints and DNA repair systems. We recently showed that the non-pou domain-containing octamer-binding protein (NONO, also known as p54NRB) contributes to the intra-S phase checkpoint activation following UVC irradiation [2].

NONO is an RNA binding protein that belongs to the *Drosophila behavior/human splicing* (DBHS) family of multifunctional proteins, including also paraspeckle component 1 (PSPC1) and splicing factor, proline- and glutamine-rich (SFPQ), which are localized into the nucleus and are involved in various aspects of RNA metabolism. NONO, which is located on chromosome Xq13.1, contains two tandem RNA recognition motifs (RRMs) and has a role in RNA processing [3, 4]. In particular, NONO can regulate transcription, forming either repressive or activating complexes and function in transcript splicing, polyadenylation, stabilization, localization and transport [4]. NONO has been also involved in the retention of hyperedited RNA in the nucleus [5], and in coupling the circadian clock to the cell cycle [6]. NONO seems able to bind also DNA and, consistently, it has been found localized onto chromatin, in sub-nuclear bodies called paraspeckles and in DNA damage-induced foci [3]. Indeed, NONO is involved in DNA repair and functions both in non-homologous end joining and homologous recombination pathways [4, 7–9]; it binds the PARP1-generated poly ADP-ribose structures at the damage sites [10]; is implicated in the cell response to both double-strand breaks [8, 11, 12] and to UVC-induced DNA damage [2].

NONO deregulation occurs in different tumour types, such as papillary renal carcinoma in which it has been found subjected to a chromosomal inversion generating a NONO/TFE3 fusion protein [13]. Moreover NONO has been found mutated in small intestine neuroendocrine tumours [14] and altered in breast, prostate and colon cancer [15–18]. Interestingly, NONO has been proposed as a factor underlying melanoma development and progression [19]. In particular, NONO is strongly expressed in melanoma samples compared with normal tissues and in melanoma cell lines compared with normal melanocytes. However, not all melanoma cell lines showed a clear correlation between mRNA and protein expression leading the authors to suggest the existence of post-transcriptional mechanisms of NONO regulation [19]. Having previously shown that NONO silencing impairs the intra-S phase checkpoint and checkpoint kinase 1 activation upon irradiation both in melanoma cell lines and in human cervix carcinoma cells, here we aimed to investigate the mechanisms of NONO regulation at the transcriptional and post-transcriptional level following exposure to UV radiations.

## RESULTS

### Analysis of *NONO* mRNA identifies putative HUR and mir320a binding sites

**Figure 1:**
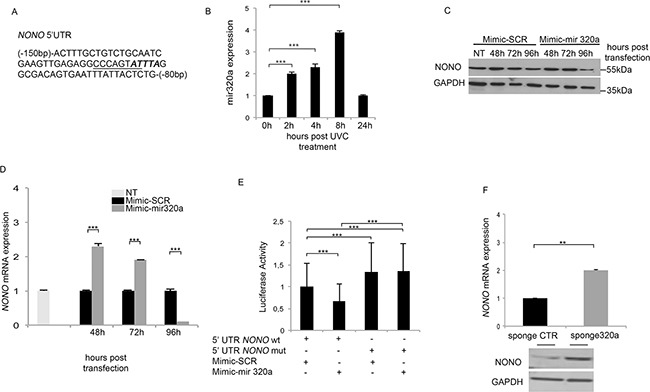
Mir320a regulates *NONO* mRNA expression **A.** A portion of *NONO* 5′ UTR (NM_001145408) including the putative HUR (bold italics) and the overlapping mir320a (underlined) binding sites. **B.** Mir320a expression in HeLa cells upon exposure to 10J/m^2^ UVC rays was evaluated at the indicated time points by real time RT-PCR. The 5S rRNA expression levels were used as a normalization control. Data are reported as fold change of miR320a expression relatively to the control (0h UVC). Statistically significant differences between the treated cells and the control cells were evaluated by Anova/Dunnett (*** *p*<0.001; n=3). Error bars denote relative S.D. **C.** NONO protein expression levels were evaluated by Western blot at the indicated time points following HEK-293 transfection with 50nM of mimic-SCR or mimic-mir320a. GAPDH expression was analyzed as a loading control. A representative blot of three independent experiments is shown. NT, non transfected. **D.** *NONO* mRNA expression was evaluated at the indicated time points following HEK-293 transfection of mimic-SCR or mimic-mir320a by real time RT-PCR. *NONO* mRNA levels were normalized to those of the β-actin gene and reported as fold change values compared to the non-transfected cells. Statistically significant differences between various conditions were evaluated by Anova/Dunnett (*** *p*<0.001; n=3). Error bars denote relative S.D. **E.** Luciferase assay of HEK-293 cells transfected with the pmirGLO-5′UTRwt and mutated plasmids in presence of mimic-SCR or mimic-mir320a. The luciferase values were normalized to those of *Renilla* activity, as an internal control. Statistically significant differences between various conditions were evaluated by Anova/Tukey (*** *p*<0.001; n=3). Error bars denote relative S.D. **F.** NONO mRNA and protein expression was evaluated by real time RT-PCR (upper panel) and Western blot (lower panel) 48 h after HeLa cell transfection of either the sponge320a or the CTR vector without UVC treatment. The result is representative of three independent experiments. Statistically significant differences between various conditions were evaluated by Student *t*-test (** *p*<0.01; n=3). Error bars denote relative S.D.

Sequence analysis of *NONO* mRNA, through the Segal lab online software (http://genie.weizmann.ac.il/), led to the identification of a putative microRNA320a (mir320a) binding site, which overlaps with one of two AU-rich elements (AREs) within the 5′ UTR (Figure 1A). Interestingly, the ubiquitous RNA binding protein HUR (also named ELAVL1) regulates target mRNAs by binding their AREs [20] and *NONO* has been previously identified as an HUR potential target by ribonucleoprotein immunoprecipitation and microarray analysis [21]. Whereas HUR role in post-transcriptional regulation upon stress conditions, including exposure to UV rays, is well established [20], the role of mir320a in the cell response to UV has not been investigated.

### *NONO* is a bona fide target of mir320a, which is induced upon exposure to UVC

So, to investigate the possible involvement of mir320a in response to UVC radiations and its possible consequences on NONO regulation, we first exposed HeLa cells to 10J/m^2^ of UVC and analyzed mir320a expression at different time points. UVC radiation treatment induced a rapid up-regulation of mir320a (Figure 1B). To assess whether *NONO* mRNA is a bona fide target of mir320a we transfected HEK-293 cells with either a mimic-mir320a or a mimic-SCR and monitored NONO protein levels. Mir320a overexpression induced NONO protein decrease 96 hours upon transfection (Figure 1C), which was mirrored by a reduction of *NONO* mRNA levels at the same timepoint (Figure 1D). Following mir320a ovexpression, however, at earlier timepoints we detected an increase in *NONO* mRNA suggesting that mir320a might lead to either an mRNA accumulation following translation block or interfere with its synthesis/stability before achieving its repressive function, which will have to be further dissected. To demonstrate that mir320a acts directly through the predicted site onto *NONO* mRNA, we cloned either the wt mir320a binding region within *NONO* 5′ UTR or a mutated (mut) form into the pmirGLO vector containing a luciferase reporter. As expected, whereas the mimic-SCR did not affect luciferase values of neither wt or mut 5′ UTR, the overexpression of mir320a reduced *NONO* wt 5′ UTR luciferase expression without altering the activity of the mut construct (Figure 1E). This mut binding region showed higher basal levels of luciferase activity compared with its wt counterpart probably owing to an impaired binding of endogenous mir320a (Figure 1E). To test this hypothesis, we transfected in HeLa cells a sponge320a expressing vector to reduce the endogenous mir320a levels. Forty-eight hours after transfection, HeLa cells showed a reduced expression of mir320a with respect to a scrambled sequence (Supplementary Figure S1A) and a concomitant increase in the basal luciferase activity of wt *NONO* 5′ UTR (Supplementary Figure S1B), further supporting the finding that mir320a targets *NONO* mRNA in the putative 5′ UTR site. In addition, we investigated the impact of mir320a silencing on NONO levels in absence of UVC treatment. The reduction of endogenous mir320a determined NONO mRNA and protein increase compared with the sponge CTR (Figure 1F).

### HUR regulates *NONO* mRNA stability in response to UV radiations

**Figure 2:**
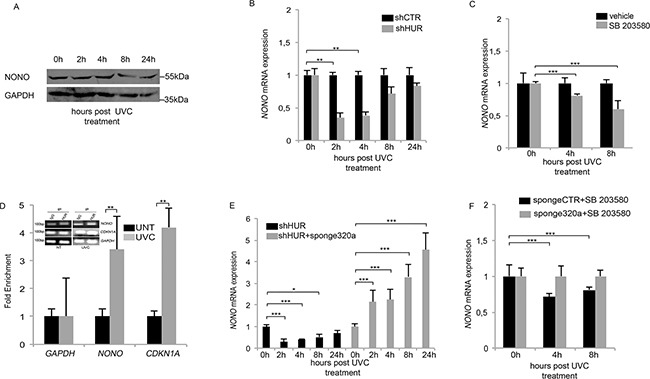
HUR protects *NONO* mRNA from mir320a-mediated degradation **A.** NONO protein levels in HeLa cells were evaluated at the indicated time points following exposure to 10J/m^2^ of UVC rays by Western blot. GAPDH was used as a loading control. A representative blot of three independent experiments is shown. **B.** HeLa cells were transfected with either the control shCTR or the shHuR vector and after 48h exposed to 10J/m^2^ UVC rays. *NONO* mRNA expression levels were analyzed at the indicated time points by real time RT-PCR and normalized to those of the β-actin gene. Statistically significant differences between various conditions were evaluated by Anova/Dunnett (** *p*<0.01; n=3). Error bars denote relative S.D. **C.** HeLa cells were pre-treated with the p38 MAPK inhibitor (SB203580) for 1h, exposed to 10J/m^2^ of UVC rays and incubated with SB203580 until collection at the indicated time points. *NONO* mRNA levels were analyzed by real time RT-PCR and normalized to those of the β-actin gene. The result is representative of three independent experiments. Statistically significant differences between various conditions were evaluated by Anova/Dunnett (*** *p*<0.001; n=3). Error bars denote relative S.D. **D.** RNA immunoprecipitation assay in HeLa cells exposed to 10J/m^2^ UVC rays followed by 4h of incubation. Real time RT-PCR was performed to quantify the amount of *NONO* mRNA bound to HUR. IgG was used as a negative immunoprecipitation control. *CDKN2A*, encoding the p21 cell cycle inhibitor, was used as a positive control whereas GAPDH was used as a normalization control. UNT, untreated. The result is representative of three independent experiments Statistically significant differences between various conditions were evaluated by Student *t*-test (** *p*<0.01; n=3). Error bars denote relative S.D. **E.** HeLa cells were transfected with either shHUR alone or in combination with the sponge320a vector and exposed to 10J/m^2^ UVC rays. *NONO* mRNA levels were analyzed by real time RT-PCR at the indicated time points and normalized to those of the β-actin gene. Statistically significant differences between various conditions were evaluated by Anova/Dunnett (* *p*<0.05; ** *p*<0.01; *** *p*<0.001; n=3). Error bars denote relative S.D. **F.** HeLa cells were transfected with the sponge CTR or sponge320a vector, pre-treated with SB203580 for 1h followed by exposure to 10J/m^2^ UVC rays and incubated for the indicated time points. *NONO* mRNA levels were analyzed by real time RT-PCR at the indicated time points and normalized to those of the β-actin gene. The result is representative of three independent experiments. Statistically significant differences between various conditions were evaluated by Anova/Dunnett (*** *p*<0.001; n=3). Error bars denote relative S.D.

So, considering that *NONO* is a bona fide target of mir320a, which is induced upon exposure to UVC, we explored whether *NONO* mRNA and protein levels were modulated following cell exposure to 10J/m^2^ of UVC. Surprisingly, but consistently with the crucial role of NONO in mediating the UVC-induced DNA damage response (DDR), UVC treatment did not change NONO expression pattern neither at the RNA (**not shown and see shCTR in Figure 2B**) or at the protein level (Figure 2A). We therefore wondered whether HUR, which regulates the function of many mRNAs involved in cell proliferation and DNA repair [22, 23], could have a role in the stabilization of *NONO* mRNA. So, we transfected HeLa cells with an HUR-targeting shRNA (shHUR) expressing vector, which effectively reduced HUR mRNA and protein levels compared with the control sequence (shCTR) (Supplementary Table S1, Supplementary Figures S1C and S1D), and exposed them to 10J/m^2^ UVC. HUR silencing indeed reduced relative *NONO* mRNA expression as early as two hours after irradiation (Figure 2B) without effect onto the untreated cells, indicating that HUR contributes to *NONO* mRNA stabilization upon exposure to UVC rays. Likely *NONO* mRNA reduction following HUR silencing is due to mir320a upregulation, which indeed peaks at two hours under these conditions (Supplementary Figure S1E).

HUR function is regulated by various phosphorylation events that modulate its ability to bind target mRNAs [24]. In particular, p38 mitogen-activated protein kinase (p38 MAPK) in response to γ radiations and UVB, has been shown to phosphorylate HUR favouring its cytoplasmic localization and mRNA target stabilization [25, 26]. So, we used a chemical inhibitor of p38 MAPK, SB203580, to block the endogenous HUR activity and we monitored the effect of UVC radiations onto *NONO* mRNA. HeLa cells were pre-treated with 10μM of SB203580 followed or not by 10J/m^2^ of UVC rays. Interestingly, the chemical inhibition of p38 MAPK reduced *NONO* mRNA level, which seemed consistent with a reduced HUR binding activity (Figure 2C). Indeed, through RNA immunoprecipitation (RIP) in HeLa cells, we found that HUR effectively binds the endogenous *NONO* mRNA in response to UVC rays. In fact, following exposure to 10J/m^2^ UVC and four hours of incubation, HUR binding to *NONO* mRNA increased three times with respect to the untreated cells (UNT) (Figure 2D), similarly to another HUR target, the cell-cycle inhibitor p21, described to be stabilized by HUR in response to UV radiations [23], which served as positive control. Overall our data indicate an important role of HUR in the stabilization of *NONO* mRNA upon exposure to UVC radiations.

### HUR protects *NONO* mRNA from degradation by the DNA damage induced mir320a

To assess whether HUR regulates *NONO* mRNA stabilization by interfering with mir320a-mediated degradation, we transfected HeLa cells with shHUR in presence or not of sponge320a and we measured the relative *NONO* mRNA expression upon UVC rays treatment. As a result, the reduction of endogenous mir320a reverted the effect of *NONO* mRNA downregulation induced by HUR silencing (Figure 2E). Finally, we pre-treated with 10μM of SB203580 the HeLa cells expressing or not sponge320a and we demonstrated how the chemical inhibition of HUR activity was able to revert the mir320a-mediated *NONO* mRNA downregulation (Figure 2F). Overall our data show that HUR binds *NONO* mRNA in response to UV rays, and protects it from mir320a action, which is consistent with previous findings showing that HUR binding within the untranslated region of its target mRNA, nearby to miRNA binding sites, competes with the mirRNAs action [27].

### p53 regulates mir320a transcription in response to UV radiation

**Figure 3:**
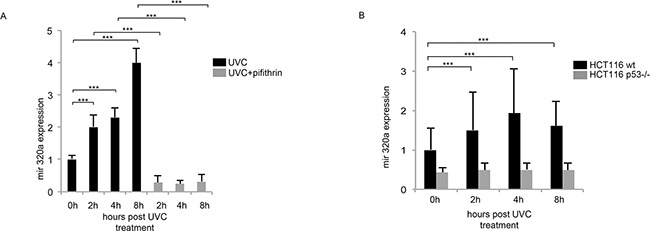
Mir320a induction in response to UVC radiations depends on p53 **A.** HeLa cells were pre-treated with 25μM of the p53 inhibitor pifithrin-α for 2h followed by exposure to 10J/m^2^ UVC rays. Mir320a expression levels were analyzed by real time RT-PCR at the indicated time points. The 5S rRNA expression levels were used as a normalization control. Statistically significant differences between various conditions were evaluated by Anova/Tukey (*** *p*<0.001; n=3). Error bars denote relative S.D. **B.** HCT116 wt and HCT116 p53−/− were exposed to 10J/m^2^ UVC radiation and mir320a levels were measured by real time RT-PCR at the indicated time points. The 5S rRNA expression levels were used as a normalization control. Statistically significant differences between various conditions were evaluated by Anova/Dunnett (*** *p*<0.001; n=3). Error bars denote relative S.D.

**Figure 4:**
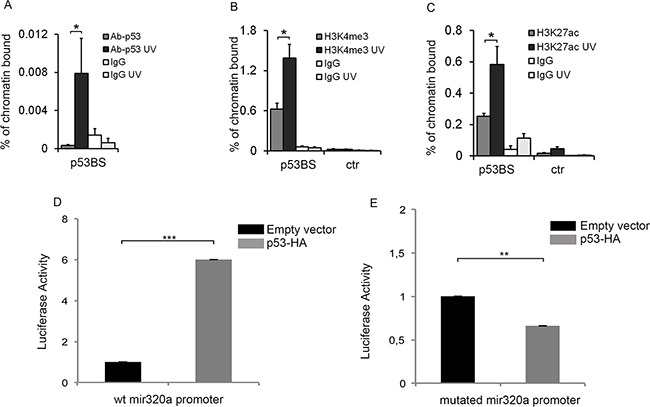
p53 directly regulates mir320a transcription in response to UVC radiations Chromatin immunoprecipitation (CHIP) assay was performed in HeLa cells 2h after exposure to 10J/m^2^ UVC by using antibodies specific for p53 **A.** (* *p*<0.05; n=3), H3K4me3 **B.** (* *p*<0.05; n=3), H3K27Ac **C.** (* *p*<0.05; n=3) or IgG as a negative control. Real time PCR was performed with p53BS amplifying primers. The GAPDH promoter region was used as control. Oligonucleotide sequences are listed in Supplementary Table S1. Statistically significant differences between various conditions were evaluated by Student t-test. Error bars denote relative S.D. **D.** HeLa cells were transfected with a pGL3-mir320a promoter wt in combination with pCEFL-HA (empty vector) or p53-HA. The activity of the luciferase reporter was evaluated 48h after transfection. *Renilla* values were used as a normalization control. The result is representative of three independent experiments. Statistically significant differences between various conditions were evaluated by Student t-test (*** *p*<0.001; n=3). Error bars denote relative S.E. **E.** Luciferase activity of the mutated pGL3-mir320a promoter was evaluated in HeLa cells 48h after transfection with pCEFL-HA or p53-HA. *Renilla* values were used as a normalization control. The result is representative of three independent experiments. Statistically significant differences between various conditions were evaluated by Student t-test (** *p*<0.01; n=3). Error bars denote relative S.E.

Given p53 role as a key regulator of the G1/S checkpoint in response to UV radiations [28], we evaluated its possible involvement in mir320a up-regulation in response to UV rays. We pre-treated for two-hours HeLa cells with 25μM of the p53 inhibitor pifithrin-α [29, 30] before treatment with 10J/m^2^ UVC. Interestingly, p53 chemical inhibition blocked mir320a up-regulation upon UV exposure (Figure 3A). To further assess whether mir320a increase depended on p53, we used wt and p53−/− colon carcinoma cell lines (HCT). Consistently with the previous experiment, UV treatment induced a time dependent up-regulation of mir320a also in wt HCT cells whereas, in p53−/−HCT mir320a expression levels did not change upon exposure (Figure 3B). To explore the possibility that p53 could directly regulate mir320a transcription, we searched through the MatInspector Software [31] possible p53 binding sites located upstream the mir320a gene. We identified eight putative p53 binding sites within an approximately 4,6 kb-long region (Supplementary Table S2). Through a preliminary chromatin immunoprecipitation-qPCR (CHIP-qPCR) assay (data not shown), we tested the possible p53 binding to these regions and then focused on two sites within the 22245703 and 22247836 chromosomal region (NC_000008.11), which showed either high or no p53 binding, defined as p53BS and ctr respectively. CHIP-qPCR analysis of HeLa cells one hour after treatment with 10J/m^2^ UVC showed that p53 indeed binds selectively p53BS following DNA damage stimuli with respect to the control antibody (Figure 4A). To confirm that the region upstream the mir320a gene can effectively function as a promoter, we performed CHIP-qPCR assays using specific antibodies against the histone H3 trimethylated on lysine4 (H3K4me3) and the histone H3 acetylated on lysine 27 (H3K27Ac), to distinguish chromatin signatures of active promoters [32]. Our data indicate that upon UVC induction H3K4me3 and H3K27Ac mark p53BS but not the ctr region, suggesting that the identified p53 binding site lies within a region compatible with an active promoter (Figure 4B,C). Accordingly, using ENCODE data [33] strong H3K4me3 and H3K27ac signals were detected in the genomic region comprising the p53BS in human keratinocytes, and in HeLa-S3, consistent with the presence of an active promoter of the mir320a gene (Supplementary Figure S2).

Finally, we cloned a 880bp-region upstream to the mir320a gene, including a wt or mutated p53BS, into a luciferase reporter vector that we transfected in HeLa along with a p53 expressing vector or the empty vector as a control. p53 ectopic expression resulted able to activate luciferase expression driven by the wt mir320a promoter region (Figure 4D) but not by the mutated one (Figure 4E).

### Mir320a silencing impairs cell viability and ATP production in response to UV radiation

**Figure 5:**
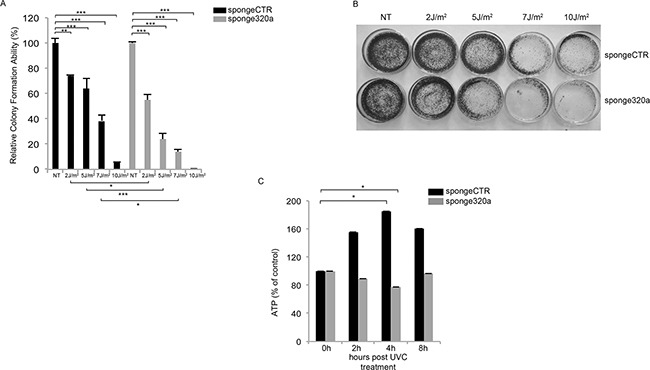
Mir320a silencing impairs cell viability and ATP production in response to UVC radiation **A.** Colony formation assay was performed in HeLa stably expressing a sponge-control or a sponge320a vector. 500 HeLa cells were treated with UVC rays at the indicated doses and stained with crystal violet upon incubation for 10 days. Statistically significant differences between various conditions were evaluated by Anova/Tukey (* *p*<0.05; ** *p*<0.01; *** *p*<0.001; n=3). Error bars denote relative S.E. A representative image of each condition is shown in **B. C.** HeLa cells were exposed to 10J/m^2^ UVC followed by incubation at the indicated time points. ATP values were normalized against protein content. Statistically significant differences between various conditions were evaluated by Anova/Dunnett (* *p*<0.05; n=3). Error bars denote relative S.E.

To investigate the biological effect of mir320a downregulation in the long term, we generated HeLa cells stably silenced for mir320a using a sponge320a plasmid. Further confirming that mir320a has indeed a crucial role in the cell response to UV radiation, we found that mir320a stable silencing was able to induce a radiation sensitive phenotype in HeLa cells treated with different doses of UVC followed by incubation for ten days. UVC treatment reduced wt HeLa cell viability to a lesser extent compared with the sponge320a silenced cells (Figure 5A, 5B).

Consistently, mir320a knockdown has been reported to sensitize cells to H_2_O_2_-induced oxidative stress. In response to oxidative stress, and in other contexts, mir320a was described to regulate glycolysis, the initial step of glucose catabolism [34]. The interplay between the cellular metabolic response and DNA damage is still poorly understood, however, inborn errors in DDR pathways in both human syndromes and mouse models typically show defects in energy metabolism [35]. So, to explore mir320a effects in this context we exposed sponge320a stably silenced cells to 10J/m^2^ UVC and we measured the ATP levels at the indicated time points, as a readout of the DNA damage metabolic response given that UV rays were shown to modulate ATP values [36]. Interestingly, UVC rays induced an up-regulation of ATP production in the control cells, whereas mir320a silencing impaired ATP production suggesting a possible role of mir320a in sustaining the metabolic response to DNA damage (Figure 5C).

## DISCUSSION

Genomic instability is a well-recognized hallmark of cancer [37]. UV radiations are among the main environmental challenge to genomic stability [38] and the primary risk factor for skin cancer, including melanoma [39]. Proteins involved in RNA metabolism act as key players in the DDR [40]. Among these, we previously found NONO to be involved in the regulation of the cell response to UV. NONO silencing impaired both HeLa and melanoma cell response to UV [2]. Interestingly, NONO was found overexpressed in malignant melanomas, in which it was suggested to be post-transcriptionally regulated [19].

Here, we set out to explore the mechanisms underlying NONO regulation in response to UV rays.

By sequence analysis, we identified a putative mir320a binding site into the 5′ UTR of *NONO* mRNA, overlapping a ARE element, which is a putative HUR binding site. HUR is an RNA binding protein with a crucial role in the post-transcriptional control of gene expression upon different stress cues, including UV [24]. HUR binds a large set of target RNAs bearing U or AU-rich sequences in their 5′ and 3′ UTRs leading to their stabilization, increased or decreased translation throughout different mechanisms [20, 41], including competitive or cooperative interactions with microRNAs [42]. Interestingly, a previous screen identified *NONO* as a potential HUR target [21]. Whereas HUR role in orchestrating the DDR is established, the role of mir320a in the UV-induced DDR is unknown. Based on all these considerations, we first assessed mir320a expression upon UV exposure and then we analyzed the interplay among *NONO*, mir320a and HUR in this setting.

We found that UVC irradiation triggered a rapid increase of mir320a expression in HeLa cells and, indeed, *NONO* is a bona fide mir320a target because the ectopic expression of a mimic mir320a was able to reduce both its mRNA and protein levels 96 hours following transfection, consistent with other reports [43]. We further confirmed that mir320a was acting by binding the predicted site into *NONO* 5′ UTR. In fact, by cloning this region, or a mutated form, in a reporter vector, we showed that a mimic mir320a could reduce luciferase activity when challenged with the wt site, but not with the mutated site, containing vector. Similarly, a sponge320a was able to increase the luciferase activity of the wt site containing vector through reduction of endogenous mir320a levels. As the sponge320a increases endogenous NONO levels in unperturbed conditions, it is likely that mir320a is involved in the maintenance of NONO steady state levels.

Following exposure to UVC radiations, however, *NONO* mRNA and protein levels remained constant despite the high mir320a induction. Although surprising this finding appeared consistent with the crucial role of NONO in the regulation of the UVC-induced DDR that we previously identified [2] and prompted us to investigate HUR function in this context.

HUR silencing decreased *NONO* mRNA as early as 2 hours upon UVC treatment but had no effect in untreated cells suggesting that HUR contributes to increase *NONO* mRNA stability following stress cues. Moreover, *NONO* decrease upon HUR silencing was likely dependent on mir320a, which also peaked at 2 hours upon UVC treatment under these conditions probably reflecting the fact that HUR silencing impacted on a wide network of players involved in the DNA damage response, including p53 itself [44].

HUR itself is tightly controlled and its activity depends on its sub-cellular localization and phosphorylation status [20, 24, 45]. In particular, upon treatment with various DNA damaging agents p38 MAPK-mediated phosphorylation induces HUR cytoplasmic localization and mRNA target stabilization [24]. Consistently, the pharmacological inhibition of p38 MAPK reduced NONO expression level both in basal conditions and upon exposure to UVC radiations, which could suggest a reduced binding of HUR and consequent impairment of its positive effect of *NONO* stability.

Indeed a RIP assay confirmed that HUR binding to *NONO* mRNA increases following exposure to UVC similarly to another HUR target, *CDKN1A*, which encodes the cell-cycle inhibitor p21 and is a key regulator of the DDR [23, 25].

To ascertain whether HUR binding to NONO mRNA protects its target from mir320a-mediated degradation, we analyzed NONO mRNA expression in HUR silenced cells upon UVC treatment and transfection of the sponge320a vector. Silencing mir320a restored NONO stability ‘complementing’ HUR absence. Similarly, silencing mir320a also restored NONO stability following p38 MAPK inhibition, not only upon UVC treatment but also under basal conditions, suggesting that mir320a and p38 MAPK might regulate the normal physiological levels of NONO whereas HUR binding seems UVC-dependent. Overall, our data suggest that NONO might be part of the HUR-coordinated RNA operon unleashed in response to DNA damage [46], which includes p53 [44] and also its target p21 [23]. Interestingly, our data add NONO to the list of genes that are targeted by HUR through the binding of their 5′ UTR, such as p27 [47], IGFIR [48], and HIF1α [49]. Whereas HUR inhibits p27 and IGF1R translation and induce HIF1α translation, here we found that HUR binding through the NONO 5′ UTR protected its target from the mir320-mediated degradation, providing a new example of competitive regulation of mRNAs by HUR and miRNAs acting at a proximal site [42].

We then wondered whether UV-induced mir320a up-regulation could be part of the p53-orchestrated response to genotoxic stress. We found that the UVC-dependent mir320a increase was abrogated both in HeLa through chemical inhibition of p53 and in HCT cells devoid of p53. Moreover, mir320a pattern of expression upon UVC treatment is consistent with the expression/activation of p53 previously reported with an increase at 6 and 12 hours and reduction at 24 hours [50]**.** In silico analysis of the mir320 locus identified putative p53 binding sites and our CHIP-qPCR data demonstrated not only that p53 effectively binds the predicted p53BS upstream the mir320 gene, indicating a direct role of p53 in UVC-dependent mir320a upregulation, but also that this region lies within an active promoter. Consistently, ENCODE data reveals strong signals of H3K4me3 and H3K27ac, two markers known to identify active regulatory regions including promoters [51, 52], in the genomic region upstream of miR320a, which includes the p53BS. Altogether, our ChIP data demonstrated that both H3K4me3 and H3K27ac signals in the mir320a promoter are increased upon UVC treatment, indicating that the promoter activity of this region is responsive to UV radiation-induced p53 action. Finally, analysis of the activity of such promoter region, upstream of a luciferase reporter, showed that p53 was able to trigger reporter activation through the predicted binding site but not through its mutated form.

Mir320a acts as a tumour suppressor in different cancer types and its downregulation correlates with chemoresistance in colon [53], breast [54] and prostate cancer [55]. Two studies so far showed that mir320a is negatively regulated by the ETS1 transcription factor [34, 54] and positively regulated by the CREB1 transcription factor in cervical cancer cells upon starvation [56]. The promoter regions described comprise also the p53 binding site herein identified. Despite p53 and ETS1 seem to have different effects on mir320a, as well as on other reciprocally regulated promoters [57], Ets1 is a key component of a UV-responsive p53 transcriptional activation complex in ES cells [58]. So, it will be interesting to assess whether also ETS1 has a role in p53-mediated mir320a regulation upon UVC exposure.

**Figure 6:**
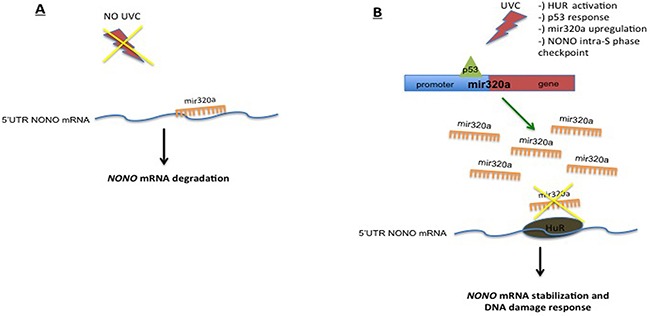
Schematic diagram depicting the p53–mir320a–HUR–NONO pathway model triggered upon exposure to UVC radiations

Overall our findings showed that, upon exposure to UVC, p53 induced mir320a expression while HUR protected NONO from mir320a-mediated degradation (see Figure 6 for a schematic diagram depicting the p53–mir320a–HUR–NONO pathway model*)*. However, what is the role of mir320a in the cell response triggered by p53 upon UVC-induced DNA damage remains to be defined. So, to assess the relevance of mir320a contribution to the DDR, we analyzed the effect of different doses of UVC radiations on HeLa cells stably silenced for mir320a. We found that mir320a stable silencing sensitized HeLa cells to UVC-induced DNA damage. Interestingly, we also found that upon UVC treatment mir320a silenced cells failed to up-regulate ATP levels. A previous report showed that UV treatment was able to induce ATP levels in rat fibroblasts and, in particular, the switch from anaerobic to oxidative phosphorylation was key to trigger cell death pathways [36]. As mir320a has been shown to reduce glycolysis by direct targeting key enzymes of this process [34], we can speculate that the p53-induced mir320a upregulation upon irradiation might underlie the complex p53 response, which includes the regulation of metabolic pathways — such as glycolysis restriction and oxidative phosphorylation enhancement — that is crucial for the activity of p53 in determining cell fate decisions following DNA damage [59].

Overall our findings reveal a new mechanism whereby HUR protects NONO for mir320a-mediated degradation in response to UVC-induced DNA damage. Moreover our data identify a new component within the complex network of players underlying the DDR adding mir320a to the list of p53-regulated targets upon genotoxic stress.

## MATERIALS AND METHODS

### Cell culture and irradiation

HeLa, HEK-293 and HCT116 cell lines were purchased from American Type Culture Collection; HCT116 cell lines were provided by Prof. Colantuoni (University of Sannio, Italy) [60]. All cell lines were grown in RPMI1640, except HEK-293 which were cultured in DMEM, supplemented with 10% foetal bovine serum (FBS), penicillin (100U/ml), streptomycin (100μg/ml), 2mM glutamine.

UV irradiation was performed, at the indicated doses, using the UVC 500 UV Crosslinker (GEHealthcare Life Sciences, Italy).

### Plasmids construction and transfection

Mimic-mir320a and mimic-SCR were purchased from ThermoScientific and transfected into HEK-293 cells through the siPORT Transfection Agent (ThermoScientific, Italy). The 5′ UTR encompassing the target sequence for miR320a onto *NONO* (NM_001145408) was amplified and cloned into *Pme*I and *Xba*I sites of pmirGLO Dual-Luciferase miRNA Target Expression Vector (Promega, Italy) (Supplementary Table S1). To generate *NONO* 5′ UTR mutated in the putative mir-320a binding site, (mut, C592G and C593G) we used the QuikChange® Site-Directed Mutagenesis Kit (AgilentTechnologies, CA, USA) and specific primers (Supplementary Table S1). To silence mir320a and HUR, we cloned into *BamH*I and *Hind*III sites of the p*Silencer* 5.1 vector (ThermoScientific) a sponge320a and a short-hairpin RNA targeting HUR (shHUR), (Supplementary Table S1). A commercial non-targeting-shRNA (shCTR) was purchased from ThermoScientific. HeLa cells were transiently transfected with Attractene Transfection Reagent (QIAGEN, Italy). The 880 bp region of chromosome 8 containing the p53 binding site upstream the mir320a locus (mir320a/p53-binding-site) (NC_000008.11=22244962–22248662nt), was amplified from human genomic DNA and cloned into *Sac*I and *Xho*I sites of pGL3-BASIC (Promega) (Supplementary Table S1). Mutations in the p53-binding site were introduced with specific primers through the QuikChange Kit (Supplementary Table S1).

### RNA isolation and quantitative RT-PCR

Total RNA was extracted from HeLa, HEK-293, HCT116 wt and p53(−/−) cells using Trizol (ThermoScientific). All samples were treated with DNaseI (ThermoScientific). For mRNA expression analysis, 500ng of total RNA were reverse-transcribed using Superscript III (ThermoScientific). All qRT-PCR reactions were performed in a 7900HT fast-RealTimePCR system (Applied Biosystem) using SYBR Green real-time PCR master mix (ThermoScientific). To quantify mir320a levels, total RNA was reverse-transcribed using the Universal cDNA Synthesis kit and analyzed using primer sets for mir-320a and for small 5S rRNA (all from Exiqon, Denmark). qRT-PCR for miRNA expression was performed using the Sybr Green master mix (Exiqon). Primers and PCR conditions are listed in Supplementary Table S1. RT-PCR data are shown as histograms reporting the fold of change of mRNA or miRNA mean expression ± relative s.d., relatively to the control at the basal level (0h of UVC treatment or untransfected). The mRNA or miRNA expression values are calculated by the 2^^−ΔΔCt^ method, relatively to controls (β-actin and 5S rRNA, respectively). Statistical analysis was performed on the ΔCt values as specified below.

### Luciferase assays

HEK-293 cells were cultured in 24-well plates and transfected with pmirGLO-*NONO* 5′ UTR (2μg) and 50nM mimic mir320a or mimic SCR with Lipofectamine2000 (ThermoScientific). Cell lysates were prepared 48h after transfection using the Dual-Luciferase Reporter Assay System (Promega) and luciferase activity was measured through the Victor X2 Multilabel Plate Reader (PerkinElmer, Italy). Luciferase values were normalized to those of *Renilla* activity, used as an internal control. HEK-293 cells were transfected with the p*Silencer*-sponge320a and pmirGLO-*NONO* 5′ UTR with X-tremeGENE DNA Transfection reagent (SigmaAldrich, Italy). HeLa cells were transfected with the pGL3 mir320a/p53-binding-site for 48h and then exposed to UV rays only or in combination with pifithrin-α. The combination experiments were carried out performing a 2h pre-treatment with 25μM pifithrin-α before irradiation.

### Immunoblots

Protein lysates and Western blot analyses were carried out according to standard procedures. Protein lysates were prepared at 4°C in 50mM HEPES pH7.5, 1% (vol/vol) Triton X-100, 150mM NaCl, 5mM EGTA, supplemented with protease and phosphatase inhibitors (ThermoScientific). Proteins from the cleared lysates were quantified and subjected to SDS-page. Antibodies against NONO (c-17, SC-23249), GAPDH (FL-335, SC-25778) and HUR (3A2, SC-5261), all from SantaCruzBiotechnology (SCBT, Germany), were used at 1:500 dilution.

### RNA immunoprecipitation (RIP)

HUR RIP was performed as previously described [61]. Briefly, HeLa cells were grown in 10×150mm plates up to 90% confluence. Cell extracts were resuspended in NT2 buffer (50mM Tris HCl pH7.5, 150mM NaCl, 1mM MgCl2, 0.05% NP40, 10mM Ribonucleoside Vanadyl Complex (New England Biolabs, MD, USA), 0,25U/ml RNaseOUT (ThermoScientific), 2mM DTT, 30mM EDTA supplemented with a protease inhibitor cocktail (SigmaAldrich) chilled at 4°C. Equal amounts of proteins (5mg) were pre-cleared for 60′ at 4°C using Protein G-Sepharose beads (PGS) (ThermoScientific) and extensively washed, while 15μg of the anti-HUR(3A2) antibody (sc-5261) or isotype control IgG1 (BD Biosciences, CA, USA) were pre-coated onto PGS beads rocking at 4°C for 16h. Then, 100μl of 50% (v/v) pre-coated beads were added to the lysates and tumbled overnight at 4°C. Beads were then pelleted and washed, followed by proteinase K treatment for 30′ at 55°C. Immunoprecipitated RNAs were extracted with Trizol and treated with DNaseI for 30′ at 37°C. cDNA was obtained as previously detailed. RIP-qPCR conditions and primers are listed in Supplementary Table S1.

### Chromatin immunoprecipitation (ChIP) assay

Chromatin immunoprecipitation (ChIP) was performed as previously described [62]. Briefly, HeLa cells were grown up to 90% of confluence in 10×150 mm plates, fixed with 1% formaldehyde 10′ at 37°C. Cells were washed twice with ice-cold PBS, ice-scraped and lysed in SDS lysis buffer. Chromatin was sheared by sonication to generate 200-1000bp DNA fragments followed by centrifugation at 14,000 rpm for 15′. For each immunoprecipitation, the NT and UV samples were pre-cleared for 1h at 4°C with Protein A-Sepharose beads (PAS) (ThermoScientific) and incubated at 4°C with 2μg of p53-D01 antibody (SCBT) or isotype control IgG1. Immunocomplexes were washed twice with 1ml TE pH8.0, cross-linking was reverted by adding 200mM NaCl and heating the samples at 65°C overnight. Samples were then treated with proteinase K (20μg), following addition of Tris pH7.0 (20μl) and 0.5 M EDTA (10μl), and incubated at 42°C for 45′. DNA was recovered by phenol-chloroform extraction and ethanol precipitation. DNA pellets were resuspended in water (50μl). For ChIP-qPCR 2μl were used per reaction and enrichment was calculated by comparison with 1% of the corresponding input sample. Data are reported as mean ± s.d. of three independent experiments. α-p53 DO1 (sc-126) and normal rabbit IgG (sc2021) were supplied by Santa Cruz Biotechnology. α-H3K3me3 (ab8580) and α-H3K4Ac27 (ab4729) were supplied by Abcam and normal isotype control mouse IgG1 (777273) were supplied by BD Biosciences. PCR conditions and primers are listed in Supplementary Table S1.

### ATPlite assay

ATP concentration was monitored using the ATPlite detection assay system (PerkinElmer). Briefly, HeLa spongeCTR and sponge320a were exposed to 10J/m^2^ UVC rays followed by incubation for the indicated time points. The ATP value was measured through the luciferase activity with the Victor X2 Multilabel Plate Reader (PerkinElmer) and normalized by the proteins content.

### Colony formation assay

For clonogenic assays, 500 cells were seeded in 60 mm plates and exposed to the indicated different doses of UVC. Two weeks after, colonies were fixed with methanol and stained with crystal violet.

### Statistical analysis

The results were expressed as the mean ± standard deviation (S.D.) or standard error (S.E.) and derived from three independent experiments, as indicated. Statistical analyses were performed using the GraphPad Software. Student *t* test was used to analyze the differences between two experimental conditions. Statistically significant differences between the means of multiple matched groups were evaluated by one-way Anova with either Dunnett post-test, to compare all data versus control, or Tukey post-test, to compare all pairs of data. *P* values are indicated by asterisks with * *p* <0.05 was considered statistically significant.

## SUPPLEMENTARY MATERIALS FIGURES AND TABLES




