# Supplementary material for: HUR protects NONO from degradation by mir320, which is induced by p53 upon UV irradiation
Source: Oncotarget. 2016 Nov 1;7(47):78127–39. doi: 10.18632/oncotarget.13002 (PMC5363649; doi:10.18632/oncotarget.13002)
Supplement: Supplementary file 1 [file oncotarget-07-78127-s001.pdf]

HUR protects *NONO* from degradation by mir320, which is induced by p53 upon UV irradiation

SUPPLEMENTARY FIGURES AND TABLES

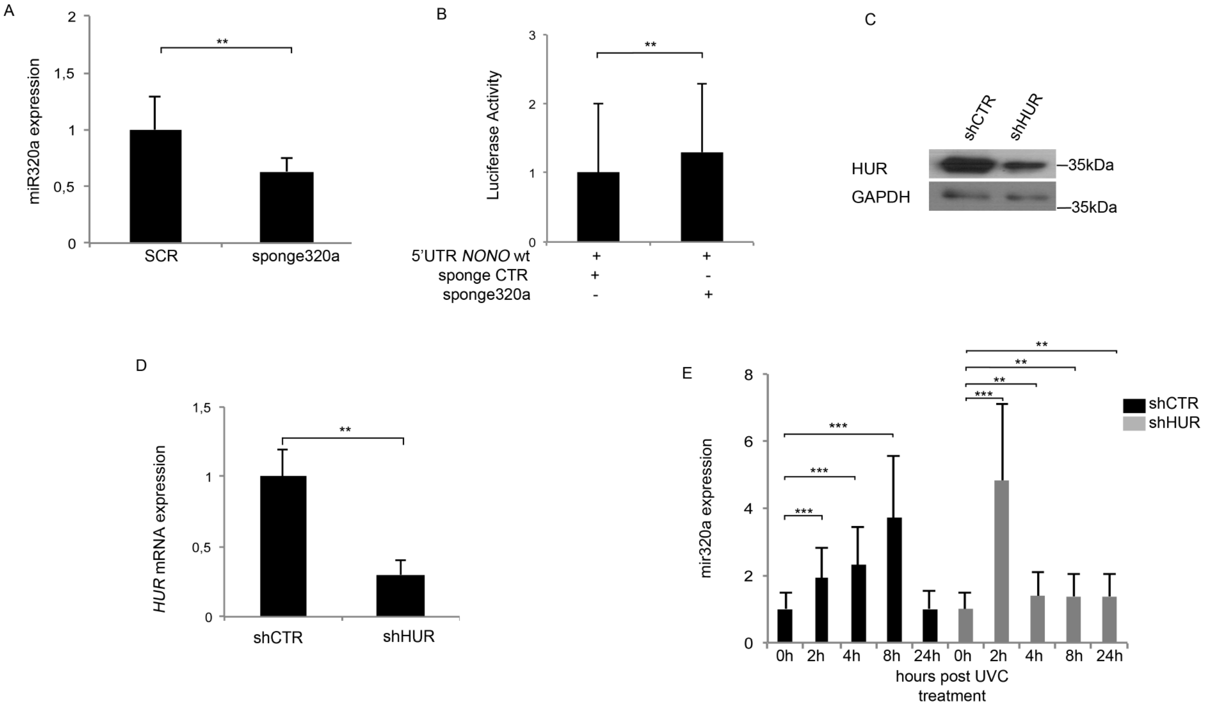

Supplementary Figure S1: A. Mir320a expression was evaluated through real time RT-PCR following transfection of HeLa cells with the sponge-control or sponge320a. GAPDH was used as a normalization control. Statistically significant differences between various conditions were evaluated by Student t-test (** $p<0.01$; n=3). Error bars denote relative S.D. **B.** HeLa cells were transfected with the pGL3-5'UTR *NONO* wt in combination with the sponge CTR or sponge320a. After 48h of transfection a luciferase assay was performed and *Renilla* values were used as a normalization control. Statistically significant differences between various conditions were evaluated by Student t-test (** $p<0.01$; n=3). Error bars denote relative S.E. **C.** Western blot analysis to assess the silencing efficiency of the shHUR plasmid respect to the shCTR in HeLa cells. GAPDH was used as a protein loading control. A representative blot is shown. **D.** *HUR* mRNA expression was evaluated through real time RT-PCR to assess the silencing efficiency of the shHUR plasmid respect to shCTR in HeLa cells. GAPDH was used as a normalization control. Statistically significant differences between various conditions were evaluated by Student t-test (** $p<0.01$; n=3). Error bars denote relative S.D. **E.** Mir320a expression was evaluated through real time RT-PCR 48h following transfection of HeLa cells with either shHUR or shCTR expressing vectors and upon treatment with 10J/m^2 at the indicated timepoints. Statistically significant differences between various conditions were evaluated by Anova/Dunnett test (** $p<0.01$; *** $p<0.001$; n=3). Error bars denote relative S.E.

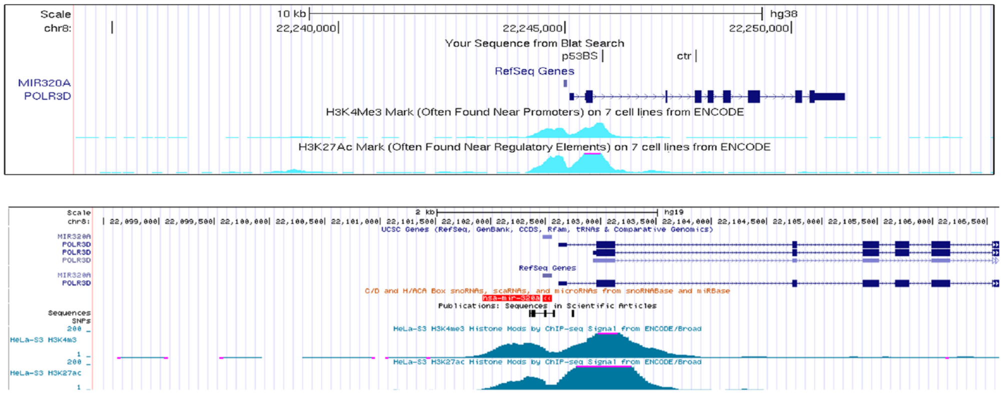

Supplementary Figure S2: The position of the p53BS and the control sites in the miR320a genomic locus are indicated. Genomic regions enriched for H3K4me3 and H3K27ac in the normal epidermal keratinocytes (NHEK) and HeLa-S3 cell lines (top panel and lower panel, respectively) were obtained from the ENCODE project (36).

Supplementary Table S1: List of primers.

See Supplementary File 1

Supplementary Table S2: List of putative p53 binding sites

Name	Position	Sequence
Putative BS1	-68bp	GTCTGCGTGGCAGGGCCTGGGCGCC
Putative BS2	-390bp	GGCGTTTCCTTCCGACATGTTGCCTGGGAGAGAA
Putative BS3 (p53BS)	-741bp	GGGAGTCATTTCAACATCTTGTACT
Putative BS4	-1268bp	GGCCAACATGGCAAAACCCTGTGTC
Putative BS5	-2874bp	GGCCTCGCCCTCGTCCATGCCCCTC
Putative BS6	-3390bp	ATCCAGCCTGCTAGCACATGCACCTG
Putative BS7	-3843bp	AGGGGCAAGAGACAACACGTTCAAG
Putative BS8	-4290bp	CCACTACAGGTCACCACTTCCC GTCCCAGTCCCCGACTTGATTC

Note: For the analysis through Matinspector we used the region **22244962-22250043** of the mir320a locus (**NC_000008.11)** containing the putative promoter.
