# Supplementary material for: HUR protects NONO from degradation by mir320, which is induced by p53 upon UV irradiation
Source: Oncotarget. 2016 Nov 1;7(47):78127–39. doi: 10.18632/oncotarget.13002 (PMC5363649; doi:10.18632/oncotarget.13002)
Supplement: Supplementary file 2 [file oncotarget-07-78127-s002.docx]

**Supplementary Table 1: List of primers**

| **Amplicon** | **Forward primer sequence**  **(5'-3')** | **Reverse primer sequence**  **(5'-3')** |
| --- | --- | --- |
| **5’UTR NONO wt** | AATTGTTTAAACCAGGCGCAGTGCAGGACTGCT | CCGGCTCTAGATTTTGCACCCTTGAGAAGTATTTC |
| **5’UTR NONO mut** | CAATCGAAGTTGAGAGGCGGAGTATTTAGGCGACAGTG | CACTGTCGCCTAAATACTCCGCCTCTCAACTTCGATTG |
| **Sponge 320a** | GATCCTCGCCCTCTGGTCCAGCTTTTAATTTCGCCCTCTGGTCCAGCTTTTTTTTTTGGAAA | AGCTTTTCCAAAAAAAAAAGCTGGACCAGAGGGCGAAATTAAAAGCTGGACCAGAGGGCGAG |
| **shHuR** | GATCCGAACACGCTGAACGGCTTGAGGTTCAAGAGACCTCAAGCCGTTCAGCGTGTTCTTTTTTGGAAA | AGCTTTTCCAAAAAAGAACACGCTGAACGGCTTGAGGTCTCTTGAACCTCAAGCCGTTCAGCGTGTTCG |
| **NONO (NM_007363.4) RT-PCR** | CGCCACCACCGCCAATACC | TCGCCTGCCTTTCCATATTTCTC |
| **HUR (NM_001419.2) RT-PCR** | ATCGTCAACTACCTCCCTCAGAACAT | GCTGTGTCCTGCTACTTTATCCCGA |
| **β-ACTINA (NM_001101.3) RT-PCR** | CAGGGCGTGATGGTGGGC | CTCGGTCAGCAGCACGG |
| **NONO (NM_007363.4) RIP** | CCTGCTAACCACATTTCCTCGTCC | TGGGTGGCTGCTCTCGTTCCTTG |
| **p21 (NM_001291549.1) RIP** | GTGGCTCTGATTGGCTTTCT | CAGCCCAAGGACAAAATAGC |
| **GAPDH (NM_001289745.1) RIP** | GTTCCAATATGATTCCACCC | CTCCTGGAAGATGGTGATGG |
| **p53BS CHIP** | GGACTGTCGTTGGGCCTCT | GGATTCTCTGCCCTTCAACC |
| **Ctr CHIP** | CAAGAAGGAGAAGCGTGAAAG | GCCCTGCTCAAAGATGGA |
| **Mir320a promoter pGL3 wt NC_000008.11:** 22245043-22245927 | GGAAGAGCTCGCCGCCTGATAAATACTGTGG | AATTAGCTAGCCTGGCTTTCATGGGTTTCTTAA |
| **p53BS mut mir320a promoter pGL3 NC_000008.11:** 22245043-22245927 | CTGGGAGTCATTTCAACATTGGTTACTCAGTACCTAGATTTGAAGGTAGGACAGACTCC | GGAGTCTGTCCTACCTTCAAATCTAGGTACTGAGTAACCAATGTTGAAATGACTCCCAG |

**PCR Conditions Denaturation Annealing Extension Cycles**

qRT-PCR 95°C, 30’’ 60°C, 60’’ 72°C, 30’’ x40

CHIP-qPCR 95°C, 30’’ 60°C, 60’’ 72°C, 30’’ x40

RIP-qPCR 95°C, 30’’ 60°C, 60’’ 72°C, 30’’ x40
